# Functional properties of glutelin from *Camellia oleifera* seed cake: Improvement by alkali-assisted phosphorylation through changes in protein structure

**DOI:** 10.1016/j.crfs.2023.100438

**Published:** 2023-01-06

**Authors:** Ningxiang Yu, Yijue Wang, Shengxin Shao, Jie Li, Mengren Li, Lizhong Zhu, Qin Ye, Weiwei Huan, Xianghe Meng

**Affiliations:** a College of Food Science and Technology, Zhejiang University of Technology, Hangzhou, 310014, Zhejiang, China; b College of Chemistry and Materials Engineering, Zhejiang A & F University, Hangzhou, 311300, Zhejiang, China; c Institute of Food Sciences, Zhejiang Academy of Agricultural Sciences, Hangzhou, 310014, Zhejiang, China

**Keywords:** *Camellia oleifera* seed cake, Glutelin, Phosphorylation, Aggregation, Solubility, *In vitro* digestibility

## Abstract

To explore the effect and its mechanism of alkali-assisted phosphorylation on the functional properties of *Camellia Oleifera* seeds cake glutelin (CSCG), CSCG was treated with different concentration of sodium trimetaphosphate (STMP, 1.0, 2.0, 3.0, 4.0, and 5%, w/v) in different pH environment (3.0, 5.0, 7.0, 9.0, and 11.0). The results showed that alkali assist improved the phosphorylation degree of CSCG, and the optimum pH value is 9.0. FT-IR and XPS confirmed the successful modification of phosphate groups on CSCG through covalent interaction. Alkali-assisted phosphorylation decreased the particle size and increased electronegativity of CSCG, as well as changed in its surface hydrophobicity, crystallinity, and intrinsic fluorescence. All these changes of protein structure triggered by alkali-assisted phosphorylation led to the improvement of water solubility, water/oil absorption capacity, emulsifying ability, foamability, and *in vitro* digestibility of CSCG. This work could provide a theoretical basis for industrial production of CSCG with excellent functional properties.

## Highlights

• Optimum phosphorylation of *Camellia* seed cake glutelin (CSCG) was achieved at pH 9.0.
• The phosphate content of CSCG increased with concentration of STMP from 0% to 3.0%.
• Phosphorylation decreased the particle size and increased the surface charge of CSCG.
• *In vitro* digestibility of CSCG were significantly increased after phosphorylation.
• Phosphorylation improved the functional properties of CSCG due to structural changes.

## Graphical abstract

**Figure:**
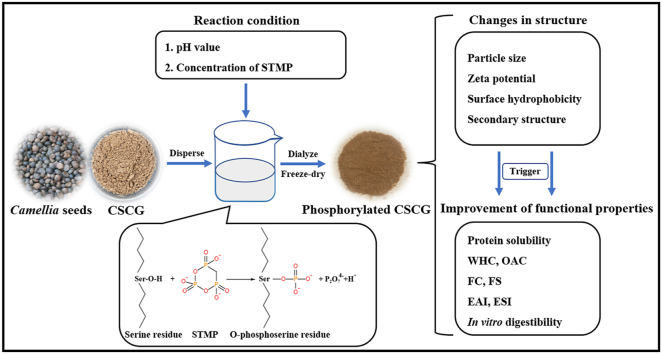

## 1 Introduction

*Camellia oleifera* seed cake (CSC) is a cheap industrial byproduct obtained after the *Camellia* oil extraction (Yao et al., 2019). The annual output of CSC in China is approximately 2 million tons, but most is discarded as waste which impact on the environment (Li et al., 2014). Notably, CSC contains 14–20% crude protein with a satisfactory composition of essential amino acids, especially lysine, leucine, and phenylalanine (Li et al., 2014). The CSC protein consists of four fractions, including glutelin, globulin, prolamin, and albumin. Among them, glutelin (CSCG) is the main fraction, accounting for approximately 40.33%, which is considered to be a source of high-quality plant protein because of its various advantages, such as low price and abundance in essential amino acids (Zhang et al., 2019; Luan et al., 2020). However, the unsatisfactory functional properties (eg. foaming, emulsification, and digestibility) of CSCG resulting from its hydrophobic and aggregate structure, limits its application in the food industry (Wang et al., 2019). Thus, modification of the structure of CSCG to improve its functional properties is necessary.

Chemical modification when using food-grade modifiers is an effective way to improve the functional properties of proteins with low cost, time savings, and convenience (Liu et al., 2012). Many chemical modification methods, such as acylation, deamidation, covalent cross-linking, oxidization, glycosylation, and phosphorylation, have been widely used in the food industry (Abedi and Pourmohammadi, 2012; Nasrabadi et al., 2021). Among them, phosphorylation has been suggested to be an economical way to enhance the functional properties of food proteins from various sources, including rice, walnut, fish, and egg (Wang et al., 2019; Yan and Zhou, 2021; Huang et al., 2018; Ren et al., 2020). Given this foundational knowledge, we aimed to improve the functional properties of CSCG through phosphorylation modification.

Phosphorylation refers to the addition of phosphate groups to the side chains of particular amino acids in a protein. The mechanism of phosphorylation occurs when phosphate groups selectively react with either a hydroxyl groups of an amino acid residue (Ser, Thr and Tyr) or specific nitrogen atoms (Lys, His imidazole ring, Arg guanidine group) to form C–O–P and C–N–P bonds, respectively (Liu et al., 2020; Hu et al., 2019). The addition of phosphate groups can enhance the negative surface charges and change the conformation of the protein, which may improve the hydration of the protein and thus increase its functional properties (Sánchez-Reséndiz et al., 2018). More importantly, previous studies have found that phosphorylation assisted by external forces such as ultrasound, microwave, and alkali can further improve the functional properties of proteins. Unfortunately, the excessive temperature caused by ultrasound and microwave resulted in the unexpected denaturation of proteins, which limited their application (Hu et al., 2022; Li et al., 2020). In contrast, alkali treatment was commonly used to accelerate the phosphorylation reactions which rarely increased the temperature of reaction system. Alkali treatment disrupted the non-covalent bonds of the protein, which led to the exposure of more sites on protein for phosphorylation reactions (Hu et al., 2019). However, little information is available on improving the functional properties of CSCG through alkali-assisted phosphorylation modification, especially for the mechanism of the improvement of functional properties of CSCG.

Here, an alkali-assisted phosphorylation method was employed to improve the functional properties of CSCG using sodium trimetaphosphate (STMP) as the food-grade phosphorylation reagent. Briefly, the effect of reaction pH value on the phosphorylation degree of CSCG was investigated to confirm the optimally alkali-assisted condition. Then, the surface and conformational characteristics of phosphorylated CSCG was discussed through analyzing its zeta potential, particle size, surface hydrophobicity (S_o_), Fourier transform infrared (FTIR) spectroscopy, X-ray diffraction (XRD), intrinsic fluorescence, and X-ray photoelectron spectroscopy (XPS). Most importantly, the effect and mechanism of alkali-assisted phosphorylation on the water solubility, water/oil absorption capacity, emulsifying property, foamability, and *in vitro* digestibility of CSCG were studied in detail. This work could provide a theoretical basis for potential utilization of CSCG modifications by phosphorylation in food industry.

## 2 Materials and methods

### 2.1 Materials

*Camellia oleifera* seed cake was purchased from local supermarket in Wuyuan city, Jiangxi, China. 8-anilino-1-naphthalenesulfonic acid (ANS) and STMP ware purchased from Aladdin Biochemical Technology Co., Ltd in Shanghai city, Shanghai, China. NaOH, HCl, phenol, and KBr were obtained from Lingfeng Chemical Reagent Co. Ltd in Shanghai city, Shanghai, China. Ammonium molybdate and sodium sulfite were purchased from Zhejiang Carl Biotechnology Co., Ltd in Hangzhou city, Zhejiang, China.

### 2.2 Extraction of CSCG

CSCG was extracted from *Camellia oleifera* seed cake according to a previous report with slight modifications (Lei et al., 2015). Briefly, after removing the albumin fraction, the cake residue was dispersed into water (solid:solvent 1:10, w/v), and the pH value of the dispersion solution was adjusted to pH 10.0 using NaOH (1 M). The suspension was stirred at room temperature for 2 h and then centrifuged at 8000 g for 10 min to obtain the supernatant. Subsequently, the pH of the supernatant was adjusted to approximately 3.0 by dropwise addition of HCl solution (2 M) and then incubated for 1 h at 4 °C. The precipitate was obtained by centrifugation at 4800 g for 10 min and then washed with 3% NaCl solution to remove globulin, 70% ethanol to remove prolamin, followed by ultrapure water three times. The resulting sample was dialyzed against distilled water for 3 days at 4 °C using dialysis tubing (molecular weight cut off: 8000–14,000 Da) and then lyophilized for further use. After that, the protein content of the sample was determined by the Kjeldahl method (N × 6.25).

### 2.3 Alkali-assisted phosphorylation of CSCG

The alkali-assisted phosphorylation of CSCG was conducted according to a previous report with slight modification (Hu et al., 2019). Briefly, CSCG was dispersed in an STMP solution at a mass fraction of 2.0%, and the pH of the suspension was adjusted to 9.0 by dropwise addition of NaOH solution (1 M) with stirring at 150 rpm. The suspension was stirred at 55 °C for 2 h and then adjusted to neutral pH by dropwise addition of HCl solution (2 M) after cooling to room temperature. The free STMP in the suspension was removed by dialyzing against deionized water for 72 h, and the deionized water was changed every 6 h. Finally, the samples were obtained by lyophilization and stored at 4 °C for further use. The effects of pH (3.0, 5.0, 7.0, 9.0, and 11.0) on the phosphorylation of CSCG were investigated at an STMP concentration of 3.0%. The effects of the concentration of STMP (1.0, 2.0, 3.0, 4.0, and 5%, w/v) on the phosphorylation of CSCG were investigated at pH 9.0. The phosphorylated CSCG (P-G) prepared at a pH of 3.0, 5.0, 7.0, 9.0, and 11.0 were named P-G-P3, P-G-P5, P-G-P7, P-G-P9, and P-G-P11, respectively. The phosphorylated CSCG (P-G) prepared at STMP concentrations of 1.0, 2.0, 3.0, 4.0, and 5% were named P-G-C1, P-G-C2, P-G-C3, P-G-C4, and P-G-C5, respectively.

### 2.4 Determination of phosphorylation degree

The total phosphate content of the samples was detected using the molybdenum blue colorimetric method with UV–vis spectrophotometer (Xiong et al., 2016). Briefly, 0.2 g of sample was suspended in a mixture solution (2 mL of sulfuric acid, 10 mL of nitric acid, and 1 mL of perchloric acid), and the suspension was digested in a digestion furnace (conditions: 120 °C for 1 h, then heated to 180 °C for 1 h and then heated to 180 °C) until it became transparent. The product was cooled and then diluted with ultrapure water to a total volume of 100 mL. Subsequently, 2 mL of the diluted sample was added to a solution containing 2 mL of 50 g/L ammonium molybdate, 1 mL of 200 g/L sodium sulfite, and 1 mL of 5 g/L hydroquinone, and then the resulting mixture was diluted with ultrapure water to a final volume of 25 mL. The sample was incubated for 0.5 h at room temperature, and its absorbance was detected at 660 nm using a UV–vis spectrophotometer. The standard curve was obtained using a KH_2_PO_4_ standard solution (0–160 μg/mL, Y = 0.0043X-0.01, R^2^ = 0.9993). To detect the inorganic phosphorus content of the sample, 0.2 g of sample was treated with 13 mL of trichloroacetic acid solution (12%) for 1 h with stirring at room temperature. Then, the suspension was centrifuged at 10,000 g for 15 min, and the inorganic phosphorus content of the supernatant was detected according to the method described above. The degree of phosphorylation was obtained by subtracting the inorganic phosphorus content of the sample from its total phosphorus content.

### 2.5 Structural characterization of phosphorylated CSCG

#### 2.5.1 Zeta-potential and particle size

25 mg of sample was dispersed into 25 mL of PBS (10 mM, pH 7.0) with stirring for 30 min at room temperature. After that, the dispersion was treated by centrifugation at 3500 rpm for 10 min to obtain the supernatant. The zeta-potential and particle size of the supernatant were measured at 25 °C using a Malvern Zeta/sizer Nano-ZS90 (Malvern Instrument Ltd., UK).

#### 2.5.2 Intrinsic fluorescence and surface hydrophobicity (S_o_)

The intrinsic fluorescence of the supernatant obtained from Section 2.5.1 was recorded with the excitation wavelength at 280 nm and emission wavelength from 315 to 400 nm using an F7000 fluorescence spectrophotometer (Hitachi Co., Japan). In order to determine the surface hydrophobicity (S_o_), the supernatant was diluted with protein concentration of 50, 100, 150, 200, and 250 μg/mL. Then, 4 mL of the diluent was incubated with 20 μL of ANS solution for10 min, and its fluorescence intensity was recorded with the excitation wavelength at 390 nm and emission wavelength 470 nm. The initial slope value of linear fitting between fluorescence intensity and protein concentration was taken as Surface hydrophobicity (S_o_).

#### 2.5.3 X-ray photoelectron spectrometer (XPS)

The surface chemical composition of the samples was studied by an X-ray photoelectron spectrometer with 25 eV pass energy (ESCALAB 250xi, Thermo Science, USA).

#### 2.5.4 Fourier transform infrared (FTIR) spectroscopy

1 mg of sample was ground into the flour with KBr (100 mg), and then placed in the cuvette. The FTIR spectra were recorded on an FT-IR spectrophotometer (Frontier, PerkinElmer, USA) from 400 to 4000 cm^−1^.

#### 2.5.5 X-ray diffraction (XRD)

3 mg of sample was ground into the flour. The X-ray diffraction (XRD) measurements were recorded from 5 to 50° at a scanning rate of 5°/min on an X-ray diffractometer (D8-Advanced, Bruker, Karlsruhe, Germany).

### 2.6 Functional properties of phosphorylated CSCG

#### 2.6.1 Protein solubility

A sample comprised of 50 mg of protein was dispersed in 5 mL of deionized water with stirring for 1 h at room temperature. After centrifugation at 6500 rpm for 15 min, the concentration of protein in the supernatant was detected by the Lowry method. Protein solubility was defined as the percentage of protein in the supernatant relative to total protein.

#### 2.6.2 Water holding capacity (WHC) and oil absorption capacity (OAC)

The WHC and OAC of the samples were measured according to our previous report (Yu et al., 2021). Briefly, 0.2 g of sample was added to 4 mL of water, and the resulting slurries were incubated for 30 min. Then, the slurries were treated via centrifugation at 2000 g for 10 min, and the volume of supernatant was recorded. The WHC was calculated according Equation (1).

(1) WHC (mL/g) = (V_2_–V_1_)/M

where V_2_ is 4 mL; V_1_ is the volume of the supernatant; and M is the mass of the sample (0.2 g).

Samples (0.2 g) were mixed with 4.0 g of sunflower seed oil, and the resulting slurries were incubated for 1 h. Then, the slurries were treated via centrifugation at 2000 g for 10 min, and the mass of free oil was recorded. The OAC was calculated according Equation (2).

(2) OAC (g/g) = (M_2_-M_1_)/M

where M_2_ is the mass of sunflower seed oil (5.0 g); M_1_ is the mass of free oil; and M is the mass of the sample (0.5 g).

#### 2.6.3 Foaming capacity (FC) and foam stability (FS)

The FC and FS of the samples were tested according to our previous method (Yu et al., 2021). Briefly, 0.5% (w/v) of the sample solution was prepared at varying pH (3.0, 5.0, 7.0, and 9.0). The solution was then homogenized at 11,000 rpm for 2 min. The FC and FS were calculated according Equations (3), (4)).

(3) FC (%) = V_b_/V_a_ × 100

(4) FS (%) = V_c_/V_b_ × 100

where V_a_ (mL) is the volume of dispersion before homogenization; V_b_ (mL) is the volume of foam at 0 min; and V_c_ (mL) is the volume of foam when left to rest for 30 min.

#### 2.6.4 Emulsifying activity index (EAI) and emulsion stability index (ESI)

The EAI and ESI of the samples were tested according to our previous method (Yu et al., 2021). Briefly, 1.0 mg/mL of the sample solution was prepared at different pHs (3.0, 5.0, 7.0, and 9.0). The solution and corn oil were mixed (3:1, v:v) and then homogenized at 11,000 rpm for 2 min to obtain the emulsion. Then, 50 μL of emulsion was pipetted from the bottom of the tube and mixed vigorously with 5 mL of SDS (0.1%, w/v). The absorbance of the mixture was measured at 500 nm by a UV spectrophotometer. The EAI and ESI were calculated according Equations (5), (6)).

(5) EAI (m^2^/g) = (2 × 2.303 × A_0_ × 100)/ [c × γ × (1-θ) × 10^6^]

where A_0_ is the absorbance of the mixed solution at 0 min; 100 is the dilution factor; c is the protein concentration (g/mL); γ is the light path, 0.01 m; and θ is the oil phase volume fraction (0.25%).

(6) ESI (%) = EAI_10_/EAI_0_ × 100

where EAI_0_ is the EAI of the emulsion at 0 min and EAI_10_ is the EAI of the emulsion following incubation for 10 min.

#### 2.6.5 In vitro digestibility

An assay to assess *in vitro* protein digestibility was performed in a simulated gastrointestinal tract as proposed by our previous study with some modification (Yu et al., 2022). Briefly, CSCG and phosphorylated CSCG were added to preheated simulated gastric fluids (0.2 mol/L NaCl, 100 mg/L pepsin (≥3000 U/mg), pH 2.0), and the final concentration of the sample was 1.0 mg/mL. After incubation for 60 min at 37 °C under continuous stirring at 100 rpm, the pH of the mixture was adjusted to 7.0, and trypsin (100 mg/L, ≥2500 U/mg) was introduced to form the simulated intestinal fluids. Then, the mixture was incubated for an additional 120 min at 37 °C under continuous stirring at 100 rpm. At different reaction intervals, 1.0 mL of the mixture was collected, heated at 90 °C for 10 min, and then centrifuged at 3000 rpm for 10 min to obtain the supernatant. The *in vitro* protein digestibility was expressed by its degree of hydrolysis (DH, %), which was detected by the ninhydrin method (Liu et al., 2021).

### 2.7 Statistical analysis

All tests were performed in parallel three times. SPSS 16.0 software was used for data analyses with one-way ANOVA. A p < 0.05 was considered to indicate a significant difference.

## 3 Results and discussion

### 3.1 Phosphorylation degree

**Fig. 1:**
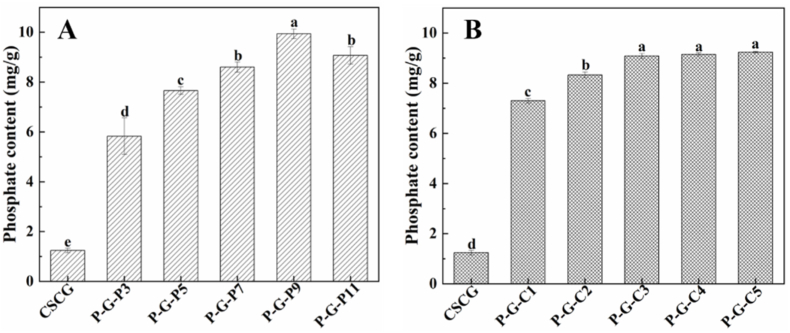
Phosphate content of CSCG and phosphorylated CSCG obtained from (A) incubation with a 3.0% STMP solution at pH 3.0, 5.0, 7.0, 9.0 and 11.0, respectively, (B) incubation in STMP solution (pH 9.0) with concentrations of 1.0, 2.0, 3.0, 4.0, and 5.0%, respectively.

Here, the use of hydrothermal phosphorylation to modify CSCG with protein content at 81.77 ± 4.06% is introduced, and the effects of reaction pH on the degree of phosphorylation is discussed. As shown in Fig. 1A, the phosphate content of CSCG was 1.24 ± 0.09 mg/g, possibly due to the phytic acids present in CSCG (Lynch et al., 1994). The phosphate content of the samples increased after incubation with a 3.0% STMP solution at different pHs, indicating the chemical addition of phosphate groups. The degree of phosphorylation first increased and then decreased with increasing of pH (3.0–11.0), and the highest phosphate content in the modified CSCG was 9.94 ± 0.19 mg/g, which was achieved at pH 9.0. This result may have occurred because the amine and hydroxyl groups within amino acid residues of CSCG were deprotonated when the reaction environment transitioned from acidic to basic, which enhanced the contact and activity between these groups and STMP (Hu et al., 2019, 2022). Meanwhile, the protein denatured when exposed to a more alkaline environment, which also influenced the degree of phosphorylation. This hypothesis can be supported by the result that as the pH increased from 3.0 to 10.0, the solubility of CSCG increased and its zeta potential changed from positive to negative (Fig. S1). However, the deprotonation of the amine and hydroxyl groups reached saturation after further increasing the pH to 11.0, and therefore, no longer contributed to additional phosphorylation. Here, pH 9.0 was chosen as the optimum condition for further experiments.

Subsequently, the effects of STMP concentrations on the degree of phosphorylation are discussed, and the results are shown in Fig. 1B. The phosphate content of P-G-C1 was 7.3 ± 0.09 mg/g, which was significantly higher than that of untreated CSCG. Interestingly, the degree of phosphorylation increased slightly and then tended to be stable with increasing concentrations of STMP from 1.0% to 5.0%. This phenomenon may be attributed to the concept that as the concentration of STMP increases, the probability of collisions with amine and hydroxyl groups in the amino acid residues of CSCG also increases, which would benefit the reaction. Since the number of amine and hydroxyl groups is fixed, the amount available to participate in the reaction is eventually exhausted, and thus, further increasing the concentration of STMP has no obvious effect on promoting the reaction (Hu et al., 2019).

### 3.2 Structural characterization of phosphorylated CSCG

#### 3.2.1 Surface characterization

**Fig. 2:**
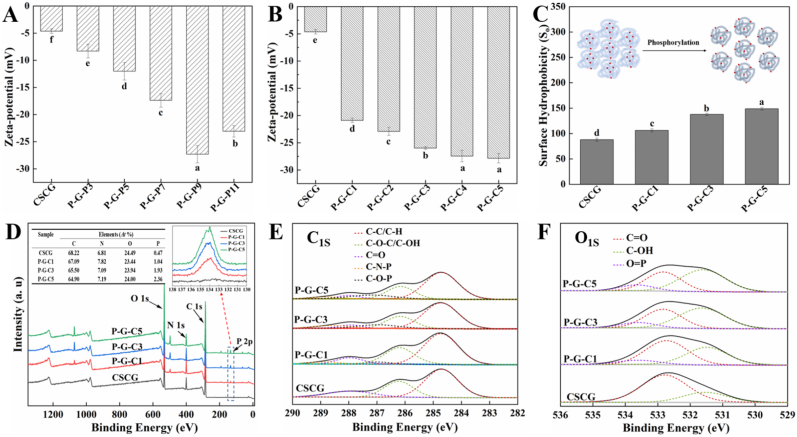
Zeta potential of CSCG and phosphorylated CSCG obtained from (A) incubation with a 3.0% STMP solution at pH 3.0, 5.0, 7.0, 9.0 and 11.0, respectively, (B) incubation in STMP solution (pH 9.0) with concentrations of 1.0, 2.0, 3.0, 4.0, and 5.0%, respectively; (C) surface hydrophobicity (S_o_) (insets: schematic representation of the changes in protein structure, the red dots represent hydrophobic groups) and (D) full-scale XPS spectra (insets: elemental analysis and spectra of P element) of CSCG and phosphorylated CSCG obtained from incubation in STMP solution (pH 9.0) with concentrations of 1.0, 3.0, and 5.0%, respectively; High-resolution XPS spectra of (E) C 1s and (F) O 1s for different samples. (For interpretation of the references to color in this figure legend, the reader is referred to the Web version of this article).

**Fig. 3:**
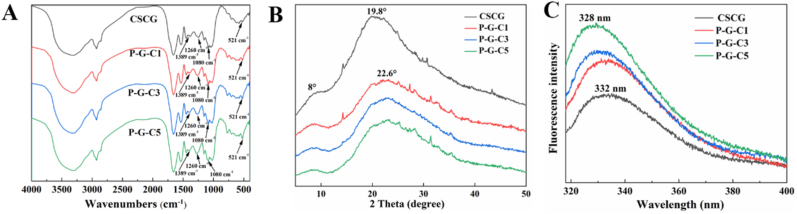
(A) FT-IR analysis, (B) XRD, and (C) intrinsic fluorescence of CSCG and phosphorylated CSCG obtained from incubation in STMP solution (pH 9.0) with concentrations of 1.0, 3.0, and 5.0%, respectively.

The effects of pH and STMP concentration on the zeta potential of phosphorylated CSCG were studied, and the results are shown in Fig. 2, Fig. 3A and B , respectively. The zeta potential of phosphorylated CSCG correlated with the degree of phosphorylation. The absolute value of the zeta potential first increased and then decreased with increasing pH from 3.0 to 11.0, and the highest absolute value of phosphorylated CSCG was 27.30 ± 1.60 mV, which was achieved at pH 9.0. Meanwhile, the absolute value of the zeta potential increased slightly and then tended to be stable with increasing concentrations of STMP from 1.0% to 5.0%. Since more negatively charged phosphate groups were introduced to the surface of CSCG, the resulted in an increased net negative charge of the molecule.

The effect of phosphorylation with different concentrations of STMP on the exposure of hydrophobic groups in CSCG was predicted using the fluorescent probe ANS. As shown in Fig. 2C, the surface hydrophobicity (S_o_) gradually increased from 87.87 ± 2.96 to 148.90 ± 2.61 with increasing of STMP concentration from 0% to 5.0%, respectively, during the phosphorylation process. This phenomenon was possibly due to several factors for which the mechanisms are shown in the inset of Fig. 2C. One possible explanation is that phosphorylation alters the form of the CSCG from that of aggregation to dispersion. This may be caused by the fact that the increased negatively charged phosphate groups on the surface of CSCG enhance the electrostatic repulsion between CSCG macromolecules (Hu et al., 2019; Aidee et al., 2018). This change greatly increased the specific surface area of the CSCG, enabling the reaction between hydrophobic groups and the ANS molecule. This hypothesis is supported by the observation that the particle size of the modified CSCG solutions rapidly decreased from 3871 nm to 123.97 nm as the concentration of added STMP increased from 0% to 5.0% (Fig. S2). Additionally, the tertiary structure of the CSCG macromolecule changes during the phosphorylation process, resulting in the inner hydrophobic amino acids of the CSCG macromolecule migrating to its surface.

The surface characterization of phosphorylated CSCG was also confirmed by XPS spectra. As shown in Fig. 2D and its inset table, the intensity of the peaks corresponding to O_1s_, N_1s_, and C_1s_ was changed after phosphorylation, suggesting alterations in the surface structure of the protein. The surface phosphate content in CSCG increased significantly after phosphorylation, and its atomic percentage increased from 0.47% to 2.36% as the STMP concentration increased from 0% to 5.0%, respectively (inset of Fig. 2D). This phenomenon indicated that the phosphate group was successfully grafted onto the surface of CSCG, consistent with the results obtained from Fig. 1B. Furthermore, the high-resolution spectra of the C_1s_ and O_1s_ regions were decomposed by peak fitting. As shown in Fig. 2E, three peaks at approximately 288 eV, 286.2 eV, and 284.7 eV were observed in CSCG, which can be attributed to the chemical states of C=O, C–O–C/C–OH, and C–C/C–H, respectively (Yan and Zhou, 2021). Notably, two new peaks at approximately 288.8 eV and 287.3 eV were observed in P-G-C1, P-G-C3, and P-G-C5, which corresponded to C–N–P and C–O–P, respectively, and suggested a change in the surface characterization of CSCG after phosphorylation (Xiong et al., 2016). Fig. 2F illustrates the spectra of the O_1s_ region for CSCG with two peaks at approximately 532.8 eV and 531.5 eV, which represented C=O and C–OH, respectively. After phosphorylation, a new peak assigned to O=P was observed at approximately 533.7 eV, and its intensity increased as the degree of phosphorylation increased, consistent with the results of Xiong and Ma, 2017).

#### 3.2.2 Conformational characterization

FT-IR analysis was used to study the conformational changes of phosphorylated CSCG. As shown in Fig. 3A, the peak occurring at approximately 1389 cm^−1^ was attributed to the stretching vibration of C–N, which became weaker after phosphorylation. This phenomenon indicated that the atoms or groups attached to the C–N were altered after phosphorylation. The shape of the absorption peak at approximately 1260 cm^−1^ changed after phosphorylation, implying the successful introduction of the P=O bond. The absorption peak at 890-1100 cm^−1^ was due to the stretching vibration of P–O. The intensity of the absorption peak occurring at approximately 1080 cm^−1^ became stronger, and the shape of the absorption peak at approximately 1023–1040 cm^−1^ changed after phosphorylation, which suggested the formation of P–O bonds. It has been reported that the stretching vibrations of P–N occurred at approximately 560–390 cm^−1^ (Wang et al., 2019). The intensity of the absorption peak occurring at approximately 521 cm^−1^ became stronger after phosphorylation, indicating that the newly added STMP was connected to the nitrogen atoms (Xiong et al., 2016). In summary, phosphorylation changed the chemical structures of CSCG by modifying their functional groups.

The conformational changes of CSCG after phosphorylation were also examined by detecting their crystallinity by XRD. As shown in Fig. 3B, the number of peaks from the samples did not change following phosphorylation, indicating that the main structure of the protein was retained. However, the diffraction peak intensity of protein at approximately 8° and 22° increased and decreased, respectively, after phosphorylation, suggesting a change in protein crystallization caused by the grafting of phosphate groups. In addition, the peak position of the protein changed from 19.8° to 22.6° after phosphorylation, which indicated a decrease in the crystal size of the protein macromolecules. This is possibly due to the destruction of the regular arrangement, reduction of the overall rigidity, and the formation of new bonds in protein macromolecules during the phosphorylation process, thus leading to the smaller crystal size (Wang et al., 2019).

The conformational changes of CSCG after phosphorylation were further investigated by monitoring intrinsic emission fluorescence spectroscopy. As shown in Fig. 3C, the fluorescence intensity of the sample increased as the degree of phosphorylation increased. This phenomenon indicated that the phosphorylation process changed the conformation of the protein, allowing more aromatic groups to be exposed on the surface of protein. It has been reported that as more aromatic groups are present on the surface of the protein, the surface hydrophobicity could increase (Ren et al., 2020), and thus, the fluorescence intensity result was consistent with the surface hydrophobicity data (Fig. 2C). Meanwhile, the maximum emission wavelength (*λ_max_*) of CSCG was blue-shifted from 332 to 328 nm with increasing concentrations of STMP from 0% to 5.0%. The blue-shift of the *λ_max_* accompanied by a decrease in fluorescence intensity, indicates that aromatic groups have migrated from the surface to the interior of the protein (Chen et al., 2013). However, the fluorescence intensity of the samples here increased with a blue-shift of their *λ_max_*. Thus, we hypothesized that the aromatic groups exposed on the protein surface did not migrate into the protein core, but instead, reacted with the phosphate groups. The blueshift of *λ_max_* is possibly because of covalent binding between proteins and other groups (Iosin et al., 2009).

### 3.3 Functional properties of phosphorylated CSCG

#### 3.3.1 Water solubility

**Fig. 4:**
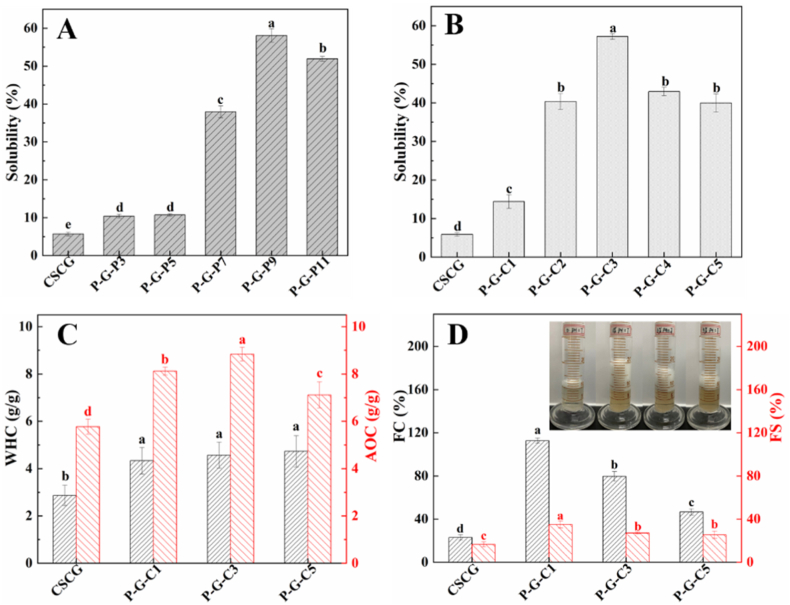
Solubility of CSCG and phosphorylated CSCG obtained from (A) incubation with a 3.0% STMP solution at pH 3.0, 5.0, 7.0, 9.0 and 11.0, respectively, (B) incubation in STMP solution (pH 9.0) with concentrations of 1.0, 2.0, 3.0, 4.0, and 5.0%, respectively; (C) water holding and oil absorption capacity (WHC and OAC) and (D) the foaming capacity (FC) and foam stability (FS) of the CSCG and phosphorylated CSCG.

As shown in Fig. 4A and B, the solubility of CSCG was very poor at only 5.86 ± 0.48%, possibly due to the low charge on the surface of the CSCG macromolecule causing a weak intermolecular repulsion, thus resulting in aggregation and precipitation. Interestingly, the solubility of CSCG increased significantly after phosphorylation. The increasing trend of the solubility was consistent with the trend of degree of phosphorylation when CSCG was phosphorylated under different pH conditions. As shown in Fig. 4A, the highest solubility of modified CSCG was 58.08 ± 1.75%, which was achieved at pH 9.0. This phenomenon may be due to the higher degree of phosphorylation of the CSCG resulting in a higher negative charge on its surface, which increases the repulsion force between CSCG macromolecules and, therefore, makes them easier to disperse into the water. Meanwhile, the hydrophilic phosphate groups on the surface of CSCG could form abundant hydrogen bonds with water molecules, which would increase the protein-water interactions and thus increase the solubility (Ren et al., 2020). However, the solubility of CSCG does not increase indefinitely with increasing phosphorylation and surface negative charge. As shown in Fig. 4B, the solubility of CSCG first increased and then decreased as the concentration of STMP increased from 1.0% to 5.0% during modification, and the highest solubility was achieved at 3.0%. This phenomenon may be due to the enhanced surface hydrophobicity of CSCG that occurs with increasing concentrations of STMP, which could weaken the protein-water interactions and cause protein aggregation. Since the surface hydrophobicity of CSCG is stronger than its surface hydrophilicity, its solubility will be reduced.

#### 3.3.2 WHC and OAC

The effects of phosphorylation at different STMP concentrations on the WHC and OAC of CSCG were studied, and the results are shown in Fig. 4C. The WHC of the untreated CSCG was 2.87 ± 0.43 g/g, which was significantly lower than that of the phosphorylated CSCG. The improved WHC of the phosphorylated CSCG was possibly because the α-helical content in the proteins decreased after phosphorylation altered the protein-water interface and enhanced their hydration (Chen et al., 2019). The WHC of the P-G-C1, P-G-C3, and P-G-C5 were similar, being 4.33 ± 0.56 g/g, 4.57 ± 0.55 g/g, and 4.73 ± 0.66 g/g, respectively, which were each higher than those of commercial soybean protein isolate (1.26 g/g) and black bean protein (2.9 g/g) (Du et al., 2018; Deng et al., 2019). In general, phosphorylated CSCG could be recommended for sticky food applications because it has a WHC ranging from 1.49 to 4.72 g/g (Deng et al., 2019). Interestingly, the OAC of P-G-C1, P-G-C3, and P-G-C5 were 8.12 ± 0.47 g/g, 8.84 ± 0.39 g/g, and 7.12 ± 0.55 g/g, respectively, which were significantly higher than that of the untreated CSCG (5.77 ± 0.32 g/g). The increased OAC of phosphorylated CSCG might be related to its increased hydrophobicity during the modification process. Notably, the phosphorylated CSCG showed superior OAC than soybean protein isolate (5.32 g/g) and wheat protein (1.7 g/g), which might be useful for fat-rich food applications, such as sauces and sausages.

#### 3.3.3 FC and FS

As shown in Fig. 4D, the FC of the untreated CSCG was only 23.0 ± 2.64%, significantly lower than those of the phosphorylated CSCG. The improved foamability of the phosphorylated CSCG is possibly attributed to its increased solubility after phosphorylation, leading to more protein macromolecule dispersion on the water-air interface. Meanwhile, increased solubility also improved the water-protein interactions on the water-air interface, which enhanced the ability of the protein to load air and accelerate the formation of more bubbles. This result was consistent with previous work showing that phosphorylation improved the foamability of rice glutelin (Wang et al., 2019). However, the foamability of the phosphorylated CSCG did not increase with increasing solubility. As shown in Fig. 4D, the FC of P-G-C1 was 112.67 ± 2.52%, which was significantly higher than that of P-G-C3 (79.65 ± 4.51%) and P-G-C5 (46.66 ± 2.79%). This phenomenon indicated that solubility was not the only factor affecting the foamability of the phosphorylated CSCG. The increased hydrophobicity of the phosphorylated CSCG with increasing phosphorylation seems to reduce their foamability, which was possibly because of the weakened water-protein interactions. As shown in Fig. 4D, the trend in FS of the sample is consistent with the trend in FC. The phosphorylation modification also significantly increased the FS of CSCG, and P-G-C1 showed the highest FS (35.08 ± 3.25%). The optimal hydrophilic and hydrophobic balance enables P-G-C1 to effectively reduce the surface tension of the water-air interface and decrease the coalescence of gas bubbles, thus maintaining the stability of foams. In addition, the effects of pH on the FC and FS of the CSCG were also investigated. As shown in Figs. S3A and 3B, the pH could affect the FC and FS of the untreated CSCG and phosphorylated CSCG, but how the pH influenced the different samples was not the same; this observation is possibly due to their different surface and conformational characteristics.

#### 3.3.4 EAI and ESI

**Fig. 5:**
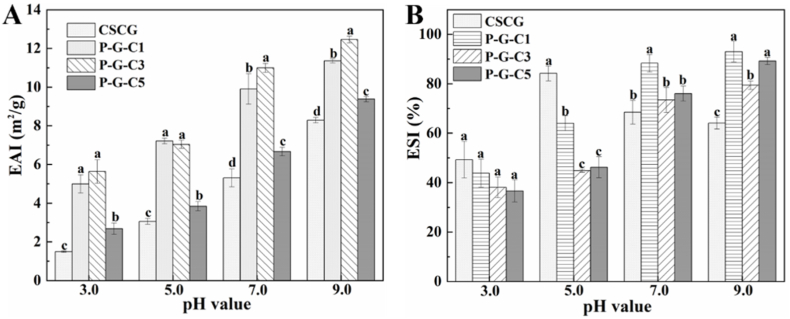
The (A) emulsifying activity index (EAI) and the (B) emulsion stability index (ESI) of the CSCG and phosphorylated CSCG at pH 3.0, 5.0, 7.0, and 9.0.

As shown in Fig. 5A, all protein samples showed minimum EAI values at pH 3.0, near their isoelectric point. This phenomenon was possibly due to the low solubility of protein near its isoelectric point weakening the adsorption capacity at the oil-water interface. The emulsifying ability of all protein samples improved as the pH increased from 3.0 to 9.0. This phenomenon is apparently due to the increased protein solubility allowing it to quickly adsorb at the oil-water interface, which easily facilitates the formation of emulsions. On the other hand, the negative charge on the protein surface increased with increasing pH, which also effectively prevented the aggregation of proteins and improved emulsification (Hu et al., 2019). Notably, the emulsifying ability of the phosphorylated CSCG significantly improved compared to that of untreated CSCG at each studied pH value. This phenomenon occurred because phosphate groups on the protein surface improved its solubility and negative surface charge. However, the increase in the emulsifying ability of CSCG is not proportional to its increase in solubility and negative surface charge. As shown in Fig. 5A, the emulsifying ability of P-G-C1 was similar to that of P-G-C3 and significantly better than that of P-G-C5 at each studied pH value. However, the solubility and negative surface charge of P-G-C1 were only 14.41 ± 1.76% and −20.9 ± 0.41 mV, respectively, which are significantly lower than those of P-G-C3 and P-G-C5. This phenomenon may be because the negative surface charge of P-G-C1 has been able to maintain protein dispersion, and dissolved protein adsorption at the oil-water interface is enough to form a uniform emulsion. Thus, the hydrophilic-lipophilic balance (HLB) of the protein becomes the determining factor in its emulsifying ability, and the HLB value of P-G-C1 seems to be more favorable for the preparation of emulsions. This phenomenon was possibly due to the more suitable unfolding and exposure of hydrophobic groups of P-G-C1 caused by the modification conditions, such as the STMP concentration, pH, and hydrothermal treatment (Wang et al., 2019).

As shown in Fig. 5B, the minimum ESI values of all protein samples were observed at pH 3.0, proportional to their EAI values. Meanwhile, for phosphorylated CSCG, the ESI increased with increasing pH consistent with an increasing trend in their EAI values as well. The pH-dependent ESI of the protein samples was possibly due to the change in their solubilities and negative surface charges. In addition, the emulsifying stabilities of P-G-C1 at pH 5.0, 7.0, and 9.0 were significantly better than those of P-G-C3 and P-G-C5, similar to the emulsifying ability results. This phenomenon might be attributed to the suitable HLB value of P-G-C1 that could form an emulsion with excessive net charge and high electrostatic repulsion, which would reduce the protein–protein interaction and prevent the aggregation of emulsion droplets (Yu et al., 2017, 2021). This hypothesis could be supported by the micrograph of emulsions demonstrating the mean droplet diameter of the P-G-C1 stabilized emulsion prepared at pH 7.0 was 2.87 ± 1.47 μm, which was significantly lower than those of the CSCG and P-G-C5 stabilized emulsions (Fig. S4).

#### 3.3.5 In vitro digestibility of phosphorylated CSCG

**Fig. 6:**
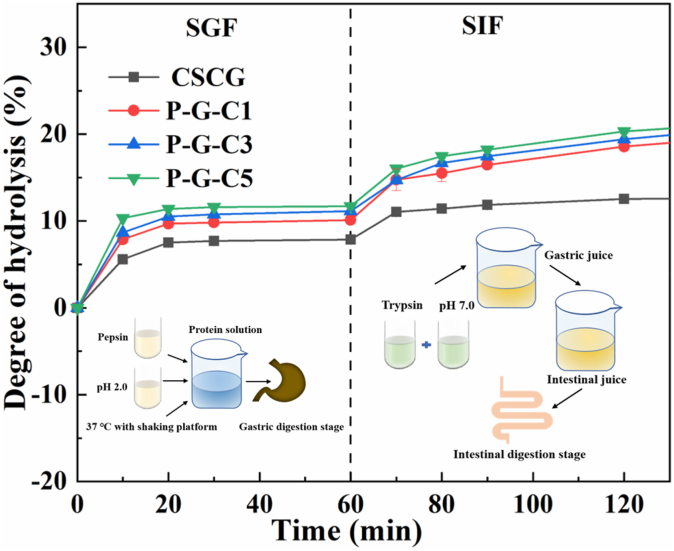
*In vitro* digestibility of the CSCG and phosphorylated CSCG: the degree of hydrolysis (DH, %) at different intervals during simulated gastrointestinal digestion.

As shown in Fig. 6, the DH value of all proteins increased during the first 20 min and then approached an equilibrium at the gastric digestion stage. Subsequently, the DH value of all proteins further increased at the intestinal digestion stage. These phenomena may possibly have occurred because the enzymatically hydrolyzable sites in the proteins were not fully exposed in the acidic environment, yet more binding sites were exposed under alkaline or neutral conditions due to changes in protein structure. The DH value of the phosphorylated CSCG increased continuously during the intestinal digestion stage, but the DH value of the untreated CSCG approached equilibrium at the later stage of intestinal digestion. This phenomenon suggests that the phosphorylation induced the CSCG to release free amino acids continuously in the intestinal environment. Notably, the DH value of the CSCG consistently increased with increasing degree of phosphorylation. This phenomenon indicated that the phosphorylation process significantly improved the digestibility of the CSCG, which was possibly due to two factors. One plausible explanation is that the form of the CSCG changed from that of aggregation to dispersion after phosphorylation (Fig. S2), which increased the contact area between the protein and digestive enzymes (pepsin and trypsin). On the other hand, it has been demonstrated that pepsin prefers to hydrolyze peptide bonds that contain the amine group of aromatic amino acids (Trp, L-Phe, and Tyr), which may also contribute to this observation (Liu et al., 2021). According to the results in Fig. 2, Fig. 3C, the amount of hydrophobic amino acids migrating from the interior of the protein to the surface of the CSCG increased along with increasing phosphorylation, resulting in the enhancement of available peptide bonds in CSCG for enzymatic cleavage. The combination of these two factors leads to an increase in the digestibility of phosphorylated CSCG. However, simulating gastrointestinal digestion *in vitro* is a screening method and thus cannot be considered an entirely accurate representation of protein digestibility (Yu et al., 2022). More suitable evaluation methods will be used to further study the effect of phosphorylation modification on the digestibility of CSCG in the future.

## 4 Conclusions

This work demonstrated that the water solubility, water/oil absorption capacity, emulsification property, foaming property, and *in vitro* digestibility of CSCG were significantly improved after phosphorylation. The structural changes in CSCG resulting from phosphorylation have been suggested to be the main reason for the improvement of its functional properties. Successfully introducing phosphate groups to the surface of the CSCG macromolecule led to an increase in the absolute zeta potential. Meanwhile, the morphology of CSCG changed from one of agglomeration to dispersion, and the inner hydrophobic amino acids of the CSCG macromolecule migrated to its surface, causing an increase in surface hydrophobicity. The changes in the CSCG structure resulted in a change in equilibrium between protein macromolecules and the external environment (water, air, oil, and other protein macromolecules), leading to an improvement in its functional properties. The results indicated that P-G-C3 showed the highest water solubility and oil absorption capacity, P-G-C1 demonstrated the highest foaming ability, and P-G-C5 underwent the greatest degree of digestion. This work can provide some guidance for the application of CSCG in food industry.

## CRediT authorship contribution statement

**Ningxiang Yu:** Conceptualization, Methodology, Writing – original draft, preparation. **Yijue Wang:** Data curation, Experiment preparation. **Shengxin Shao:** Experiment preparation, Data curation. **Jie Li:** Language polishing. **Mengren Li:** Language polishing. **Lizhong Zhu:** Data curation. **Qin Ye:** Language polishing. **Weiwei Huan:** Writing manuscript, Data curation. **Xianghe Meng:** Supervision, and Guiding the experiment.

## Declaration of competing interest

The authors declare that they have no known competing financial interests or personal relationships that could have appeared to influence the work reported in this paper.

## Data Availability

we permitted to share our data
