# Supplementary material for: Functional properties of glutelin from Camellia oleifera seed cake: Improvement by alkali-assisted phosphorylation through changes in protein structure
Source: Curr Res Food Sci. 2023 Jan 6;6:100438. doi: 10.1016/j.crfs.2023.100438 (PMC9842863; doi:10.1016/j.crfs.2023.100438)
Supplement: Multimedia component 1 [file mmc1.docx]

**Functional properties** **of glutelin from *Camellia oleifera* seed cake: Improvement by** **alkali-assisted** **phosphorylation through** **changes in protein structure**

Ningxiang Yu^a^, Yijue Wang^a^, Shengxin Shao^a^, Jie Li^b^, Mengren Li^b^, Lizhong Zhu^b^， Qin Ye^c^, Weiwei Huan*^b^, Xianghe Meng*^a^

^a^ College of Food Science and Technology, Zhejiang University of Technology, Hangzhou 310014, Zhejiang, China.

^b^ College of Chemistry and Materials Engineering, Zhejiang A & F University, Hangzhou, 311300, Zhejiang, China.

^c^ College of Biology and Environmental Engineering, Zhejiang Shuren University, Hangzhou 310015, China.

*Corresponding author: Tel: +86-0571-88320137

E-mail: vivid96@aliyun.com (W, Huan); mengxh@zjut.edu.cn (X, Meng)





**Figure S1.** Zeta potential and solubility of CSCG at different pH value.





**Figure S2.** The particle size of CSCG, P-G-C1, P-G-C3, and P-G-C5 solution.


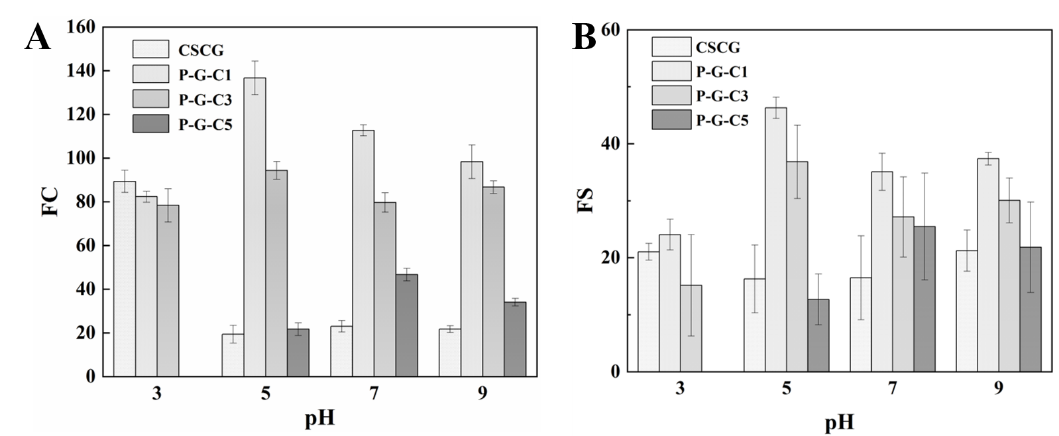


**Figure S3.** the (A) foaming capacity (FC) and (B) foam stability (FS) of CSCG, P-G-C1, P-G-C3, and P-G-C5 at pH value 3.0, 5.0, 7.0, and 9.0.


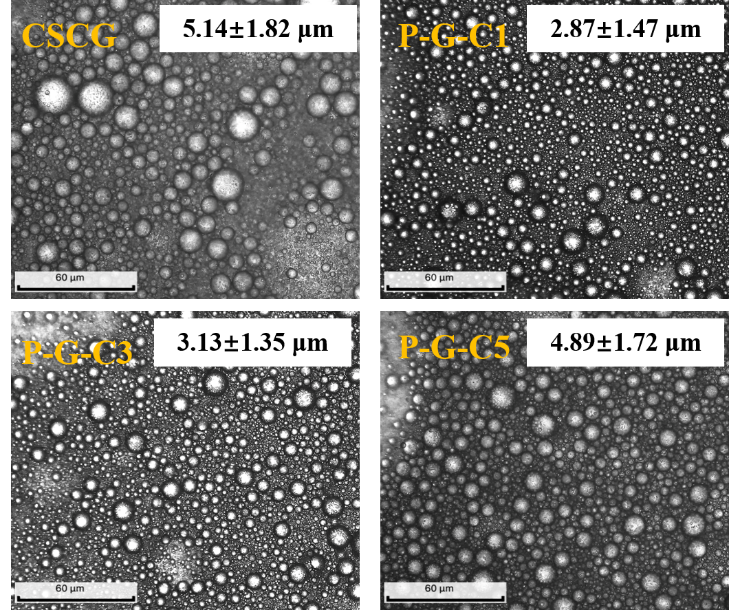


**Figure S4.** Micrograph and mean droplet diameters (inset) of emulsions prepared at pH 7.0 which were stabilized by CSCG, P-G-C1, P-G-C3, and P-G-C5.
